# Imaging biomarkers in optic neuritis: current tools and future directions

**DOI:** 10.3389/fneur.2025.1666835

**Published:** 2025-10-03

**Authors:** Ziwei Meng, Yuhong He, Kai Guo, Lin Li

**Affiliations:** ^1^Department of Ophthalmology, The Affiliated Hospital of Inner Mongolia Medical University, Hohhot, China; ^2^Department of Neurology, Mayo Clinic, Rochester, MN, United States

**Keywords:** optic neuritis, imaging biomarkers, optical coherence tomography, optical coherence tomography angiography, magnetic resonance imaging, artificial intelligence

## Abstract

Optic neuritis (ON), a central manifestation of multiple inflammatory central nervous system (CNS) disorders, has seen remarkable advances in diagnostic and therapeutic strategies due to rapid progress in imaging technologies. This review systematically summarizes recent high-quality literature focusing on the latest progress of optical coherence tomography (OCT), optical coherence tomography angiography (OCTA), magnetic resonance imaging (MRI), and diffusion tensor imaging (DTI) in ON. It further explores the integrative application and clinical value of multimodal imaging combined with immune biomarkers. Additionally, the application of artificial intelligence (AI) and deep learning (DL) in image analysis is discussed. This review highlights current innovations and proposes future directions for establishing multicenter standardized protocols, facilitating precision diagnostics, and promoting personalized management, thereby accelerating clinical translation and advancing neuroimmunological ophthalmology.

## 1 Introduction

Optic neuritis (ON) is an inflammatory demyelinating condition of the optic nerve, presenting with acute or subacute visual loss, often accompanied by pain on eye movement, and can be unilateral or bilateral (1, 2). ON may manifest either as an isolated episode or as the initial/concomitant feature of central nervous system (CNS) demyelinating disorders, particularly multiple sclerosis (MS), neuromyelitis optica spectrum disorder (NMOSD), and myelin oligodendrocyte glycoprotein antibody-associated disease (MOGAD), highlighting the clinical heterogeneity that complicates early diagnosis (3–5). Since the clinical implementation of aquaporin-4 immunoglobulin G (AQP4-IgG) testing in 2004 (6), the etiological spectrum of ON has been refined, prompting a shift from purely symptom-based to immunopathology-informed classification.

Despite advances in our understanding, ON still presents several diagnostic and management challenges. Distinct ON subtypes differ markedly in attack frequency, visual recovery, treatment response, and long-term prognosis; yet early clinical features often overlap, leading to frequent misdiagnosis (7, 8). Conventional auxiliary tests such as visual fields (VF), visual evoked potentials (VEP), and magnetic resonance imaging (MRI) often serve as exclusion tools rather than providing quantitative assessments of inflammation and axonal injury (9, 10). While high-dose corticosteroids remain the first-line treatment during acute ON episodes, imaging biomarkers capable of evaluating treatment efficacy and predicting relapse remain lacking, limiting personalized monitoring and management strategies.

In recent years, the emergence of high-resolution imaging tools such as optical coherence tomography (OCT), Optical coherence tomography angiography (OCTA), and MRI—particularly advanced sequences like diffusion tensor imaging (DTI)—has ushered ON research into a new era of quantifiable visualization (11). OCT provides micron-level precision in measuring retinal nerve fiber layer (RNFL) and ganglion cell–inner plexiform layer (GCIPL) thickness, thereby reflecting axonal and neuronal integrity (3, 5). OCTA enables non-invasive assessment of microvascular density and perfusion characteristics in the optic disc and macula, offering insights into etiological differences and visual recovery prognosis (12). MRI and DTI detect microstructural abnormalities in the optic nerve tract and associated regions, correlating strongly with clinical outcomes (13, 14). Moreover, the integration of artificial intelligence (AI) and deep learning (DL) technologies is transforming automated image analysis and feature extraction, enabling large-scale quantitative studies (15). As a result, imaging biomarkers are increasingly central to early detection, etiological classification, therapeutic monitoring, and prognostic evaluation, progressively anchoring ON management within the framework of precision medicine.

This review presents innovations in the following areas: (1) Multimodal integration: combining OCT, OCTA, MRI/DTI, and AI-based technologies to systematically compare the advantages of structural, perfusion, and microstructural imaging; (2) Synthesis of recent evidence: focusing on high-quality studies from 2023 to 2025, including differentiation between NMOSD and MS, and AI-based automated ON classification and feature extraction; (3) Feasibility of clinical pathways: proposing an integrated diagnostic and therapeutic framework based on imaging and immune biomarkers, including AQP4-IgG, MOG-IgG, and neurofilament light chain (NfL). By framing ON as a model intersection of neuroimmunology and ophthalmic imaging, this review underscores the central role of imaging biomarkers in elucidating disease mechanisms and guiding precision therapeutic strategies, thereby emphasizing their transformative potential in both clinical and research settings.

## 2 Optical coherence tomography

OCT, a noninvasive imaging modality with micrometer resolution, has become a cornerstone in assessing structural damage to the optic nerve and retina in patients with ON. By quantifying changes in the RNFL and ganglion GCIPL, OCT reveals demyelination and axonal degeneration processes. Beyond structural assessment, OCT informs disease classification, prognostication, and treatment monitoring, while facilitating integration with other imaging modalities.

### 2.1 RNFL and GCIPL thickness and visual function in ON

#### 2.1.1 Inter-eye difference and temporal dynamics

Idiopathic ON/MS-related ON. RNFL thinning typically becomes evident within the first 3 months after ON onset, with reductions averaging 15–18 μm, and correlates robustly with subsequent visual acuity recovery, underscoring its role as a sensitive biomarker for early post-acute damage (16). GCIPL thickness offers a more specific reflection of ganglion cell integrity, correlating strongly with contrast sensitivity and visual field deviations, and providing a sensitive early biomarker (17). Narrative synthesis of multiple studies suggests that inter-eye difference (IED) metrics, rather than absolute values, are especially powerful in idiopathic and MS-related ON. Thresholds of ≥4 μm GCIPL inter-eye absolute difference (IEAD) or ≥4% inter-eye percent difference (IEPD) yield AUC values approaching 0.90 across devices (18). Similar RNFL-based cutoffs reliably identify prior ON even when absolute values remain within normative ranges (19). These findings support GCIPL-based IED as a practical biomarker for early post-acute evaluation of idiopathic and MS-associated ON.

MOGAD and NMOSD. By contrast, antibody-mediated ON demonstrates distinct longitudinal patterns. In MOGAD, GCIPL decline is measurable within weeks post-ON and parallels 10-2 visual field loss; RNFL may continue to decrease up to 12 months, reflecting ongoing neuroaxonal damage or edema resolution (20, 21). In NMOSD, RNFL loss is typically more abrupt and diffuse with limited structural recovery, consistent with AQP4-IgG–mediated astrocytopathy, and GCIPL thinning is often more pronounced after attacks (22). These antibody-specific dynamics illustrate that although IED is broadly informative, its interpretation must be tailored to the underlying etiology.

#### 2.1.2 Structure–function correlation beyond high-contrast visual acuity

Beyond high-contrast visual acuity (VA), OCT metrics correlate quantitatively with more sensitive functional indices. In a prospective cohort of 88 acute ON patients, eyes with greater GCIPL thinning exhibited a mean loss of 8.5 letters on the 2.5% low-contrast Sloan chart at 6 months (*p* < 0.01), despite comparable high-contrast VA, highlighting the importance of low-contrast letter acuity (23). This underscores that contrast sensitivity and visual field indices provide more sensitive functional correlates of OCT-detected neurodegeneration.

### 2.2 Predicting ON prognosis and relapse risk with OCT

Beyond structural assessment, OCT is increasingly used to monitor treatment response and predict ON relapse. Notably, RNFL thinning > 20 μm combined with prolonged visual evoked potential (VEP) latency > 12 ms significantly increased relapse risk (hazard ratio = 2.7, *p* = 0.003) (24). Additionally, early RNFL thinning correlated with reduced retinal microvascular density, and their combination enhanced relapse prediction accuracy (25). Building on structural-functional correlations, a predictive model based on OCT-derived GCIPL metrics in traumatic optic neuropathy demonstrated that GCIPL thickness served as an independent prognostic factor for visual recovery, achieving an area under the curve (AUC) of 0.90 within 1 year (26). Further study confirmed that accelerated longitudinal GCIPL thinning over 6–12 months is strongly associated with poorer visual outcomes and increased relapse risk (27). OCT also plays a role in monitoring disease progression within the framework of No Evidence of Disease Activity (NEDA) in MS, where preserved retinal thickness correlates with sustained NEDA status and reduced relapse risk (28).

### 2.3 OCT and ON of different etiologies

Recent studies also provide disease-specific insights for MOGAD and NMOSD, highlighting structural changes along the anterior and posterior visual pathways that reflect distinct neurodegenerative patterns. OCT provides valuable information not only for prognosis but also for differentiating ON subtypes. GCIPL thinning, particularly in the parafoveal region, is more pronounced in NMOSD-associated optic neuritis (NMOSD-ON) than in multiple sclerosis–associated optic neuritis (MS-ON), and strongly correlates with cognitive and visual processing speed deficits (r = −0.62, *p* < 0.001) (23). Further studies have demonstrated that MS-ON primarily affects the temporal RNFL quadrants, AQP4-IgG + NMOSD-ON results in diffuse loss across all quadrants, and MOGAD-ON exhibits relatively symmetric but milder thinning (*p* < 0.001) (29).

In MOGAD, eyes with prior ON show significant reduction in pRNFL and GCIPL thickness, which correlates with the number of ON episodes, as well as structural atrophy along the visual pathway, including lateral geniculate nucleus (LGN) and occipital cortex, indicating pathway-specific neurodegeneration (30). In NMOSD, LGN volume is reduced after ON, associated with retinal neuroaxonal loss and optic radiation damage, but does not decline in the absence of new ON episodes, suggesting that neurodegeneration primarily follows acute attacks (22). Collectively, these findings highlight OCT’s value in capturing disease-specific progression patterns across different ON etiologies.

In line with these findings, it has been observed that despite severe acute vision loss, MOGAD-ON often retains relatively preserved GCIPL thickness (mean reduction 14 μm), in contrast to NMOSD-ON eyes, which show markedly greater loss (mean reduction 28 μm, *p* = 0.008) (31). Recent comparative reviews and cohort analyses provide consistent evidence that MOGAD-ON is frequently bilateral at onset, associated with pronounced optic disc swelling, and followed by variable but sometimes modest chronic GCIPL loss despite profound acute deficits. By contrast, AQP4-IgG + NMOSD-ON is characterized by severe and diffuse chronic pRNFL and GCIPL thinning after attacks, with average chronic pRNFL values often <80 μm, whereas MS-ON more commonly shows temporal-predominant RNFL loss (19). These modality-specific signatures support the use of OCT-guided triage for serologic testing when clinical features are ambiguous (Table 1).

**Table 1:** Comparative imaging discriminators across ON subtypes.

Imaging modality	NMOSD-ON (AQP4-IgG+)	MOGAD-ON (MOG-IgG+)	MS-ON	Idiopathic ON
OCT: RNFL/GCL thinning	Severe, often widespread	Prominent but with partial recovery	Focal thinning, especially temporal quadrant	Variable, usually mild
OCTA: Vessel density changes	Pronounced deep capillary plexus loss	Moderate superficial plexus loss	Mild vessel density decrease	Minimal changes
MRI: Lesion distribution	Longitudinally extensive optic nerve lesions (≥3 segments)	Anterior, often optic nerve head swelling	Retrobulbar, short-segment lesions	Mixed patterns
MRI: Contrast enhancement	Marked, long, continuous	Prominent, anterior	Patchy, short segment	Variable
Advanced MRI (DTI, MRS)	Reduced FA, increased radial diffusivity; metabolic changes consistent with demyelination + axonal loss	Less severe DTI changes; relative axonal preservation	Focal demyelination; subtle metabolic changes	Nonspecific
Prognosis (functional outcome)	Poor visual recovery, high recurrence risk	Often good recovery; relapses common	Intermediate recovery; associated with MS conversion risk	Usually favorable

Beyond descriptive features, newer analytic models further enhance diagnostic classification. An OCT-based model was developed that accurately distinguished diffuse RNFL and GCIPL thinning in NMOSD-ON, temporal thinning in MS-ON, and relatively preserved retinal structure in MOGAD-ON despite significant visual loss (32). Similarly, inter-eye difference (IED) metrics were shown to be useful, with GCIPL IED significantly greater in MOGAD-ON compared to MS-ON and NMOSD-ON, achieving a sensitivity of 87% and specificity of 85% for subtype discrimination (33). Finally, longitudinal MS cohorts demonstrate the prognostic dimension of OCT biomarkers, with pRNFL quantification identifying individuals at increased risk of cognitive dysfunction (23). Together, these findings underscore the capacity of OCT-derived metrics to capture both disease-specific phenotypes and long-term outcomes more comprehensively.

### 2.4 Technological advancements and future directions

High-speed swept-source OCT (SS-OCT) has been shown to significantly enhance imaging depth and speed, improving visualization of deep optic nerve structures and reducing motion artifacts, thereby enabling more accurate detection of optic disc edema and structural distortion in ON patients (34). Quantitative birefringence analysis using polarization-sensitive OCT (PS-OCT) has been reviewed, highlighting its ability to detect pre-atrophic microstructural disorganization in the RNFL, which may aid in identifying ON activity before irreversible damage occurs (35). A novel search algorithm for OCT layer segmentation has been developed, achieving highly precise delineation of retinal layers even in severely distorted optic nerves, thereby facilitating robust ON subtype classification and treatment monitoring (36). Looking ahead, the integration of advanced OCT modalities with artificial intelligence and longitudinal data analysis is expected to further enhance the early detection, classification, and individualized monitoring of ON, paving the way toward predictive and precision neuro-ophthalmology.

## 3 Optical coherence tomography angiography

With advances in imaging technology, OCTA, a non-dye, noninvasive blood flow imaging modality, has increasingly been used to investigate retinal microvascular alterations in ON and related CNS demyelinating diseases, showing remarkable potential for evaluating visual function recovery and aiding disease classification.

### 3.1 Microvascular changes in ON

OCTA has revealed significant reductions in peripapillary and macular vessel densities during both acute and chronic stages of ON, offering valuable insights beyond traditional structural metrics. Quantitative studies of demyelinating ON have shown that vessel density loss in the superior and inferior quadrants is significantly more pronounced (*p* < 0.001) and closely correlates with visual field defects (r = 0.72, *p* < 0.01) (37). Additionally, peripapillary choroidal microvasculature dropout has been associated with poor visual recovery in ON patients (*p* = 0.003), even after adjusting for RNFL thickness, suggesting that deeper vascular alterations may serve as independent prognostic biomarkers (38). Early OCTA-detected reductions in vessel density have been shown to precede structural thinning and predict long-term functional decline, particularly in patients experiencing recurrent ON episodes (25). Together, these results highlight OCTA’s potential as a sensitive, non-invasive modality for early detection, prognostication, and treatment response evaluation in ON.

### 3.2 Application of OCTA in differential diagnosis of ON in MS, NMOSD, and MOGAD

A systematic review of OCTA biomarkers in MS and NMOSD identified consistent patterns of pronounced superficial capillary plexus (SCP) rarefaction in NMOSD, with peripapillary vessel density reductions exceeding 20%, whereas MS exhibited milder changes primarily in the temporal quadrant (39). These findings reinforce the value of SCP integrity as a subtype-specific imaging biomarker. Mohammadi et al. (40) conducted a meta-analysis of OCTA data across 11 studies, reporting that NMOSD patients had significantly larger foveal avascular zone (FAZ) areas and lower parafoveal vessel densities than both MS and healthy controls (*p* < 0.001), while MOGAD patients exhibited near-normal microvascular metrics. This highlights the utility of FAZ morphology and flow indices in distinguishing NMOSD-ON from other ON subtypes. Furthermore, novel OCTA-derived indices, including vessel tortuosity and fractal dimension, have been introduced to evaluate microvascular complexity (39). Findings indicate that NMOSD-ON is associated with markedly reduced capillary regularity and impaired vascular remodeling potential, providing mechanistic insights and supporting OCTA’s role in disease classification and activity monitoring.

### 3.3 Advances in deep learning for OCTA image analysis

Recent innovations in deep learning (DL) have significantly enhanced the accuracy, efficiency, and reproducibility of OCTA analysis. Several state-of-the-art DL frameworks have been reviewed, highlighting the robustness of U-Net variants and attention-based models in accurately segmenting fine vascular structures, achieving vessel segmentation Dice coefficients exceeding 0.90 and reducing manual annotation by over 70%, thereby paving the way for standardized and fully automated ON-related vascular imaging analysis (41). A multi-view tri-alignment deep learning framework, MuTri, has been proposed to translate structural OCT into synthesized OCTA volumes with high fidelity, overcoming inter-modality variability. This model achieved over 25% improvement in cross-modality translation accuracy compared to baseline GAN architectures, enabling simulated 3D OCTA generation in cases where perfusion data may be unavailable (42). Collectively, these developments position DL as a critical tool for earlier and more precise characterization of microvascular changes in ON and related demyelinating conditions.

## 4 Magnetic resonance imaging and diffusion tensor imaging

### 4.1 Conventional MRI sequences in ON and prognostic evaluation

Conventional MRI sequences, including T2-weighted, STIR, and contrast-enhanced T1-weighted imaging, remain foundational for detecting optic nerve lesions, enhancement, and demyelination in ON and related disorders, providing essential diagnostic and prognostic information (43). Radiological predictors of visual outcome in ON have been investigated, showing that greater optic nerve lesion length and higher enhancement intensity during acute episodes are significantly associated with poorer visual recovery at 12 months (r = −0.71, *p* < 0.001), highlighting inflammatory load as a key prognostic indicator (44). Furthermore, contrast-enhanced MRI studies have shown that optic nerve enhancement lengths exceeding 17 mm are significantly correlated with initial deficits in high-contrast visual acuity and contrast sensitivity (*p* < 0.05), reinforcing the utility of MRI enhancement metrics in gauging acute disease severity and guiding early treatment decisions (45).

Although optic nerve MRI is currently optional for MS diagnosis, it provides crucial information for lesion assessment, monitoring disease progression, and predicting visual outcomes (46). Thus, conventional MRI sequences, together with emerging quantitative assessments, serve both diagnostic and prognostic roles, enhancing ON clinical evaluation.

### 4.2 DTI performance in microstructural damage of ON

DTI has emerged as a sensitive imaging modality to capture microstructural changes in the optic nerve. The use of DTI in optic neuropathies has been reviewed, highlighting that elevated apparent diffusion coefficient (ADC) and reduced fractional anisotropy (FA) in the acute phase are predictive of subsequent axonal degeneration and vision loss, supporting early DTI assessment as a reliable predictor of long-term structural damage (47). The pathophysiological mechanisms underlying optic neuritis in multiple sclerosis have been reviewed, highlighting that DTI metrics reliably reflect microstructural axonal damage and demyelination processes in the optic nerve, thus serving as essential biomarkers for disease progression (48). Quantitative spinal MRI studies have demonstrated that structural changes in the spinal cord following optic neuritis closely relate to optic nerve damage and visual impairment, suggesting broader CNS involvement detectable by advanced imaging (49). Overall, these findings support DTI as a sensitive tool for capturing microstructural changes predictive of visual outcomes in ON, offering valuable insights for tailored therapeutic interventions.

Optic nerve MRI findings have been shown to correlate with cerebral MRI changes in MS, supporting the idea that both conventional and non-conventional imaging can provide a comprehensive view of disease activity (50). The value of DTI and other quantitative MRI sequences for assessing optic nerve microstructure has been emphasized, highlighting their potential to predict long-term visual outcomes and guide early therapeutic interventions (46).

### 4.3 Manifestations and diagnostic advances in different ON etiologies

MRI features can aid in distinguishing ON etiologies. A retrospective analysis of 56 ON patients showed that MOGAD-ON more frequently presented with bilateral involvement and lesions extending to distal segments, such as the intraorbital and canalicular regions, which was distinct from MS-ON and NMO-ON (*p* = 0.006 and *p* = 0.039, respectively) (51). Furthermore, combining brain/spinal cord and optic nerve MRI features with OCT RNFL thickness enabled differentiation of MS from NMOSD/MOGAD with 95% classification accuracy (*p* < 0.001) (52).

MRI patterns, including optic nerve vs. sheath involvement, can distinguish typical (MS-related) from atypical ON (NMOSD- or MOG-IgG-related), guiding tailored treatment strategies (53). In addition, MRI features of intraocular optic nerve disorders have been summarized, highlighting the role of MRI in lesion characterization, extent assessment, and monitoring therapeutic response (54). These findings collectively highlight that MRI, when combined with advanced sequences and OCT, improves etiological classification and informs individualized management in ON.

Comparative analysis of DTI parameters in white matter tracts of MS and related disorders demonstrated that decreased axial diffusivity and fractional anisotropy strongly correlate with retinal nerve fiber layer thinning and visual evoked potential abnormalities, reinforcing DTI’s utility for early detection and longitudinal monitoring of microstructural injury (55).

### 4.4 Combined OCT and MRI studies in ON

Integration of OCT and MRI enhances understanding and management of ON by linking structural and functional changes. Enhanced depth imaging OCT was used to identify retrolaminar hyper-reflective foci in MS patients with acute ON, revealing significant associations with MRI-detected lesions (*p* = 0.000) (56). Moreover, OCT-measured pRNFL thinning in 50 patients with optic neuropathy, specifically linked to ON, was shown to complement MRI assessments, with significant correlations to lesion length (*p* = 0.01), indicating that OCT enhances MRI’s diagnostic precision by capturing retinal changes associated with optic nerve damage (57). This approach was further validated in 79 MS patients with ON, showing that incorporating OCT-assessed optic nerve data into MRI-based dissemination in space (DIS) criteria increased diagnostic sensitivity by 12% (*p* = 0.03), improving early ON diagnosis without compromising specificity, thereby highlighting the synergistic potential of these modalities (58). Overall, these findings underscore the synergistic potential of combining structural and functional biomarkers, linking retinal and optic nerve pathology to enhance both diagnostic accuracy and therapeutic decision-making, paving the way for a more holistic understanding of ON pathophysiology.

## 5 Intelligent imaging analysis: artificial intelligence and deep learning in ON

AI and DL are progressively applied in ON research, providing standardized and automated solutions for visual function evaluation, and advancing imaging analysis from descriptive to quantitative and predictive levels.

### 5.1 Inferring blood flow maps from structural OCT

A convolutional neural network (CNN) model was employed to infer superficial capillary blood flow maps from standard OCT images, achieving 85% prediction accuracy and a Dice coefficient of 0.82 across independent ON samples (59). This demonstrated that microvascular perfusion could be assessed without OCTA. Cassottana et al. (60) conducted a comparative study assessing papillary and macular blood flow using OCTA in healthy subjects and patients with various optic neuropathies, providing normative and pathological benchmarks that enhance the interpretation of inferred flow maps. Together, these approaches strengthen deep learning models’ ability to discriminate ON etiologies by integrating structural and vascular imaging data.

### 5.2 Super-resolution and multimodal fusion of blood flow–structure imaging

An enhanced OCTA post-processing pipeline using attention-guided super-resolution algorithms was introduced, significantly improving vascular detail resolution in retinal imaging, with PSNR increased by 3.9 dB and SSIM by 0.06 compared to baseline interpolation methods (61). This method enables more accurate visualization of capillary-level changes in optic neuropathies, facilitating earlier and finer diagnostic discrimination. Moreover, a multimodal deep learning framework integrating structural OCT, OCTA, and serum omics data was developed, achieving an AUC of 0.95 for distinguishing inflammatory versus ischemic optic neuropathies and predicting six-month visual outcomes with 91.2% sensitivity and 89.5% specificity (62). These advances underscore the value of multimodal fusion in enhancing ON diagnostic precision and outcome prediction.

### 5.3 Automated OCTA processing and 3D reconstruction technologies

High-resolution 3D OCT volumes combined with synthetic slice generation were leveraged to improve lesion detection in demyelinating diseases, achieving 92.3% diagnostic accuracy in distinguishing MS-related optic neuropathy from controls and enhancing visualization of peripapillary microstructural abnormalities (63). In parallel, Vision Transformer (ViT) technology was applied for 3D reconstruction of OCTA images, resulting in a 20% improvement in accuracy (64), enabling more comprehensive depiction of tissue and blood flow changes in various ON types. Additionally, AI groups have optimized segmentation strategies via reinforcement learning, reducing noise error by 15% and enhancing microvascular change detection across ON etiologies (41). Collectively, these developments position DL as a critical tool for more precise characterization of microvascular changes and overall ON imaging.

## 6 Imaging characteristics for pediatric optic neuritis

ON displays a distinct epidemiological profile compared to adults, with MOGAD-ON being more prevalent in children, accounting for 30–64% of pediatric demyelinating syndromes (65), while MS-ON and NMOSD-ON are rarer in this group and more common in adult (66). In children, OCT reveals pronounced RNFL and GCIPL thinning during acute attacks, with median peripapillary RNFL thickness reaching 164 μm due to severe swelling, but with better recovery potential (67). Concurrently, MRI shows extensive longitudinal optic nerve lesions with bilateral involvement and perineural enhancement, while OCTA highlights reduced peripapillary vessel density (e.g., 10–15% lower than age-matched controls) and widespread microvascular changes during inflammation (68). These findings contrast with adults, who exhibit greater chronic RNFL thinning (average reduction of 20–30 μm post-attack), shorter MRI lesions with higher chiasmal involvement, and more stable but diminished OCTA vascular networks reflecting persistent damage. These differences underscore the need for pediatric-specific reference standards for age-adjusted RNFL/GCIPL norms and microvascular metrics, alongside validated AI algorithms to enhance diagnostic accuracy and monitor progression, reflecting developmental variations in retinal and optic nerve anatomy.

## 7 Integration of imaging biomarkers and immunological biomarkers

**Figure 1:**
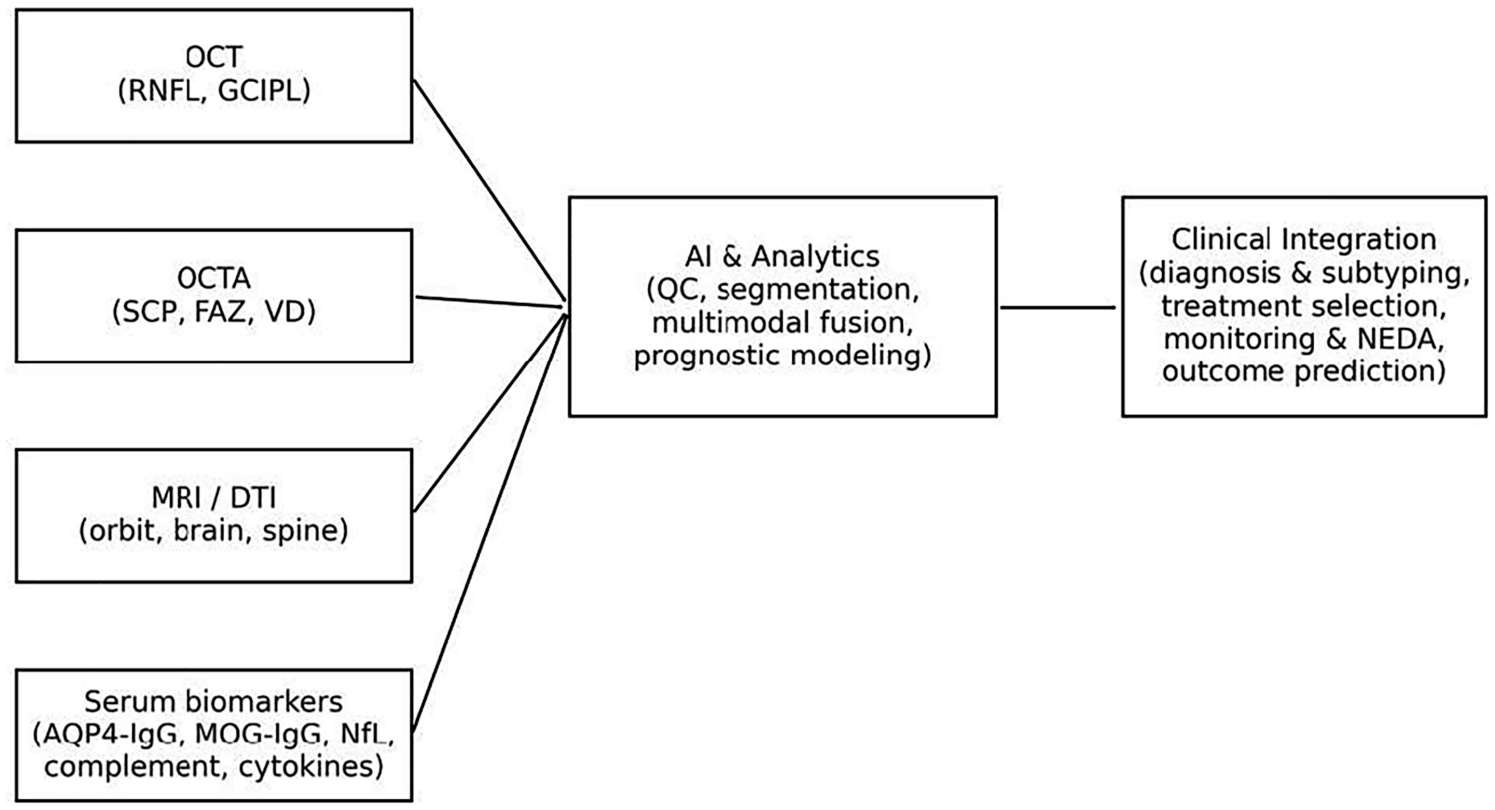
Integrated precision medicine framework for optic neuritis (ON). The schematic illustrates the integration of multimodal imaging, serum biomarkers, and artificial intelligence (AI)-driven analytics to support clinical in ON. Left panel includes structural optical coherence tomography [OCT; retinal nerve fiber layer (RNFL), ganglion cell–inner plexiform layer (GCIPL)], optical coherence tomography angiography [OCTA; superficial capillary plexus [SCP], foveal avascular zone (FAZ), vessel density (VD)], magnetic resonance imaging/diffusion tensor imaging (MRI/DTI; orbital, brain, and spinal cord assessments), and serum biomarkers [AQP4-IgG, MOG-IgG, neurofilament light chain (NfL), complement components, cytokines]. These data streams converge into AI-based analytics encompassing quality control (QC), automated segmentation, multimodal data fusion, and prognostic modeling. Outputs from this computational framework inform the right panel, representing clinical integration, including diagnosis, subtype classification, treatment selection, longitudinal monitoring, assessment of No Evidence of Disease Activity (NEDA), and prediction of visual and functional outcomes. This model emphasizes the synergistic application of imaging, immunological profiling, and computational methods to guide individualized management strategies in ON.

Recent studies highlight the synergistic value of combining imaging and immunological biomarkers in the etiological diagnosis of ON (Figure 1). Specific MRI features—such as the volume and distribution of optic nerve enhancement—have been shown to correlate significantly with serum AQP4-IgG titers in NMOSD-ON patients, suggesting that AQP4-IgG levels may directly reflect central inflammatory burden on imaging (69). Complementing this, OCTA-derived microvascular parameters were integrated with serum MOG-IgG and AQP4-IgG antibody levels, providing a combined structural–immunological assessment (70). Their multimodal approach significantly improved the differentiation between MOGAD, NMOSD, and MS, outperforming individual biomarkers alone. Overall, these findings underscore the diagnostic value of integrating immune and imaging data for accurate ON subtype classification and disease monitoring.

Emerging multi-omics studies have deepened our understanding of how immunological activity shapes imaging manifestations in ON. Elevated serum levels of complement components C3a and C5a have been shown to correlate significantly with greater RNFL thinning in NMOSD-ON, indicating that OCT-detected structural damage reflects ongoing complement-mediated axonal injury (*p* < 0.01) (71). Additionally, integration of cytokine panels with OCTA metrics has demonstrated that elevated IL-6 and TNF-*α* levels correlate with reduced peripapillary vessel density (r = −0.54, *p* < 0.001), highlighting the close link between systemic inflammation and retinal microvascular rarefaction (70). These findings support a dual-dimensional biomarker approach—combining immune indicators with imaging parameters—for improved ON subtype differentiation and dynamic disease monitoring.

For disease activity and prognosis evaluation, Serum NfL levels measured during acute ON attacks have been shown to strongly predict post-attack disability progression and correlate with structural imaging parameters, supporting their role as dynamic biomarkers of axonal damage (72). Complement components, including C1q and C3, have been highlighted as emerging predictors of disease severity and visual prognosis in ON. When integrated with structural imaging parameters, they improve sensitivity for detecting subclinical inflammation and long-term axonal damage, indicating their potential as adjunct biomarkers for monitoring chronic disease progression (73). AI-based algorithms integrating OCT and MRI features with serum immune profiles have been shown to enhance ON subtype classification and guide treatment stratification. These models achieved high diagnostic accuracy across multi-etiology datasets, demonstrating the potential of AI to combine multimodal data for precision medicine applications (74). Serum cytokine panels, including IL-6, VEGF, and miR-150, have been shown to correlate closely with OCT-derived RNFL and GCIPL measurements. Such immunological markers complement structural imaging in assessing optic nerve integrity and differentiating ON subtypes, providing a foundation for biomarker-informed clinical decision-making (70). These markers are closely associated with RNFL and GCIPL thickness changes detected by OCT, providing refined neural structural damage information across ON etiologies and complementing conventional structural imaging, thus laying a solid foundation for future multimodal biomarker validation.

## 8 Current challenges and future directions of imaging biomarkers

Despite substantial advances in imaging technologies for the diagnosis and monitoring of ON, several clinical challenges persist. Significant heterogeneity in imaging parameters arises from variations in device platforms, scan acquisition protocols, and segmentation algorithms, complicating dataset harmonization across centers and limiting multi-site validation efforts (16). This lack of standardization hinders the reproducibility and comparability of results, especially in large-scale studies.

In parallel, most current AI applications in neuro-ophthalmology face limited generalizability, as highlighted by Kenney and Requarth (75). Models are frequently trained on narrow datasets from single institutions or specific devices, leading to performance declines when applied to external cohorts. Additionally, the lack of explainable outputs and standardized clinical integration frameworks further hinders their widespread adoption in practice (75). These limitations highlight the urgent need for collaborative multi-center efforts, open-access imaging repositories, and standardized pipelines to facilitate the development of robust, generalizable AI-enhanced imaging tools. These issues highlight the importance of data sharing, external validation, and multicenter collaborative research. Only through establishing standardized workflows and open databases can cross-institutional model training and validation be achieved to ensure reliability and universality.

Looking forward, the integration of multimodal imaging with artificial intelligence is emerging as a cornerstone for enhancing diagnostic accuracy and individualized prognostication in ON. It has been demonstrated that integrating structural imaging modalities (e.g., OCT, MRI) with functional assessments such as OCTA and electrophysiological measures, supported by AI-driven analytical frameworks, allows for a more comprehensive characterization of the multifactorial pathophysiology of optic neuritis (76). It was validated that smartphone-based self-screening tools are feasible for early detection of neuro-ophthalmic disorders, underscoring their potential to expand access to real-time assessment, enable remote disease surveillance, and facilitate longitudinal monitoring beyond traditional clinical settings (77). Future interdisciplinary, multicenter, and multimodal research frameworks will be pivotal to advancing ON diagnosis and treatment toward precision medicine.

## 9 Conclusion

ON, a hallmark of central nervous system inflammatory disorders, relies critically on imaging biomarkers for accurate diagnosis, prognostic assessment, and clinical management. Advances in multimodal imaging—including optical coherence tomography (OCT), OCT angiography (OCTA), and magnetic resonance imaging/diffusion tensor imaging (MRI/DTI)—combined with AI-driven analytics and immune profiling, have transformed etiological differentiation, relapse prediction, and personalized treatment selection. Despite ongoing challenges in standardization and AI generalizability, these biomarkers now anchor the entire ON management continuum—from early detection through long-term monitoring—highlighting their pivotal role in driving precision neuro-ophthalmology.
